# Development and validation of the perceived symptom manageability scale among people living with the human immunodeficiency virus

**DOI:** 10.1186/s40359-024-01658-0

**Published:** 2024-03-25

**Authors:** Meilian Xie, Aiping Wang, Zhiyun Zhang, Kerong Wang, Yanping Yu, Xiaojing Ma, Zhaoxia Lin, Zhengli Yu, Jianxue Ke

**Affiliations:** 1 Department of Nursing, Beijing Ditan Hospital Capital Medical University, Beijing, China; 2 Department of Public Service, The First Hospital of China Medical University, Shenyang, Liaoning Province China; 3 Beijing Home of Red Ribbon, Beijing Ditan Hospital Capital Medical University, Beijing, China; 4 Department of Infectious Disease, Beijing Ditan Hospital Capital Medical University, Beijing, China; 5 Department of Quality Control, Beijing Ditan Hospital Capital Medical University, Beijing, China

**Keywords:** HIV/AIDS, Perceived manageability, Reliability, Scale development, Symptom management, Validity

## Abstract

**Background:**

“Perceived Symptom Manageability (PSM)” is essential in symptom management among people living with HIV. As a standardized assessment instrument was lacking, we developed a PSM scale for people living with human immunodeficiency virus (PSM-HIV).

**Methods:**

Data analysis was performed using the sample from HIV-designated medical institutions (*N* = 540). Psychometric testing, namely reliability and validity, is assessed by unidimensionality, internal consistency, exploratory and confirmatory factor analysis, and structural equation modeling.

**Results:**

The final version of the PSM- HIV scale contained 15 items. This scale was submitted to a principal components analysis with varimax rotation, and three factors were obtained, explained by a total variance of 63.10%. The three factors were named Cognitive-Behavioral, Affective Interaction, and Self-Attitude. The results show that the scale had high reliability, Cronbach’s α of the scale ranged from 0.71 to 0.92, and the Intraclass Correlation Coefficient was 0.88. The structural equation model supports a factor model with the acceptable fit (χ^2^/df (CMIN/DF) = 2.50, Root Mean square Residual (RMR) = 0.03, Goodness-of-Fit Index (GFI) = 0.93, Adjusted Goodness of Fit Index (AGFI) = 0.90, Normed Fit Index (NFI) = 0.93, Incremental Fit Index (IFI) = 0.96, Comparative Fit Index (CFI) = 0.96). The average variance extracted was 0.38 ∼ 0.59, and the composite reliability was 0.70 ∼ 0.91, indicating that the convergent validity of the scale is acceptable. Subjects with different stages of the disease reached significance(χ^2^ = 9.02; df = 2, *P*<0.05), meaning moderate Known-Groups Comparison Validation.

**Conclusions:**

The PSM-HIV scale is a valid instrument that measures overall attitude and belief about controlling or coping with HIV-relevant symptoms.

## Introduction

People living with the human immunodeficiency virus (PLWH) face a range of symptoms in every stage of HIV [1, 2]. The existing evidence points out that most PLWH often report more than five symptoms co-existed [3–5], including physical symptoms (such as fatigue, fever, cough, mouth ulcer… etc.), psychological symptoms (such as anxiety and depression), and cognitive symptoms (such as having difficulty in concentrating, slow react, memory loss and so on). These lead to poor adherence to ART [6, 7], poorer quality of life [8], and suicidal ideation [9]. The development of precision medicine has contributed to the rise of symptom science [10, 11], and with rapid advances in diagnostic and treatment technologies, the life expectancy of HIV-infected patients is approaching that of the general population [12, 13], meaning a long-term persistence of the symptomatic state and calling for the development of precise symptomatic care and management strategies [14]. A cross-sectional study in China reveals that the Chinese PLWH’s needs for symptom management are unmet due to their severe symptom burden, HIV-related stigma, and limited professional support from medical staff [15]. The practice of symptom management among PLWH is essential and pressing in China.

Since Spirig et al. [19] developed the Self-regulatory HIV/AIDS Symptom Management Model (SSMM-HIV), which is currently the only international theory used to guide symptom management of PLWH. Management of symptoms involves the daily decision-making of people living with HIV regarding various aspects of symptom management. This includes determining the appropriate times to reach out to healthcare providers, deciding when to take medications, and evaluating whether adjustments to exercise or diet are necessary [19]. SSMM-HIV is a a recursive model consisting of multiple concepts such as symptom experience, symptom management, social support, treatment adherence, clinical outcomes, and quality of life, which elucidates the factors influencing the quality of life and clinical outcomes in PLWH, provides new perspectives and directions for symptom management practice. In recent decades, symptom management practices based on SSMM-HIV have become a focal point of research in the HIV field [14, 16], particularly by inspiring intrinsic motivation in PLWH for symptom management, making a significant contribution to this field [17, 18]..

In SSMM-HIV, a new concept was explained in detail by the developers, namely Perceived Symptom Manageability (PSM) [20], guiding researchers and healthcare professionals to better understand the intrinsic motivation of PLWH. PSM was identified as the extent of the perceived ability to bring social and personal resources to deal with or control symptoms successfully despite difficulties, which may serve as a basis to identify not only symptoms but also areas of intervention that are of most concern to individual patients. As one of the concepts with significant value in SSMM-HIV, PSM represents the cognitive and emotional evaluation of individual symptom management endeavors by addressing the overall success of these actions as perceived by the PLWH, changing the previous understanding of symptom management from the healthcare professionals to the perceived ability or control of PLWH to self-manage and cope with symptoms in daily life. It can also create an interactive environment for medical cooperation among doctors, nurses, and PLWH [20].

In addition to this, assess the manageability, emphasizing (successful) control as the core dimension of manageability [21]. Several studies [22, 23] have indicated a potential connection between perceived control and self-efficacy, suggesting that PSM could influence self-efficacy and serve as a potential motivating factor in shaping patients’ initiatives for symptom management, clinical outcomes, and changes in quality of life. These may bring new enlightenment and inspiration for symptom management in PLWH. Nevertheless, PSM is merely a concept formed by integrating existing evidence available at the time. To successfully attain the aforementioned objectives, it becomes imperative to precisely measure PSM, thereby validating its significance and clinical contributions within the SSMM-HIV model and other associated contexts. Hence, this study will integrate established concepts, theories, and frameworks, guided by the COSMIN (Consensus-based Standards for the selection of Health Measurement Instruments) steering committee’s revised and updated COSMIN-RoB (COSMIN Risk of Bias) checklist from 2018 [24]. The fundamental goal is to address practical research questions and develop the PSM-HIV Scale by a scientific and standardized process, and evaluate its psychometric properties.

## Methods

### Setting and design

**Fig. 1:**
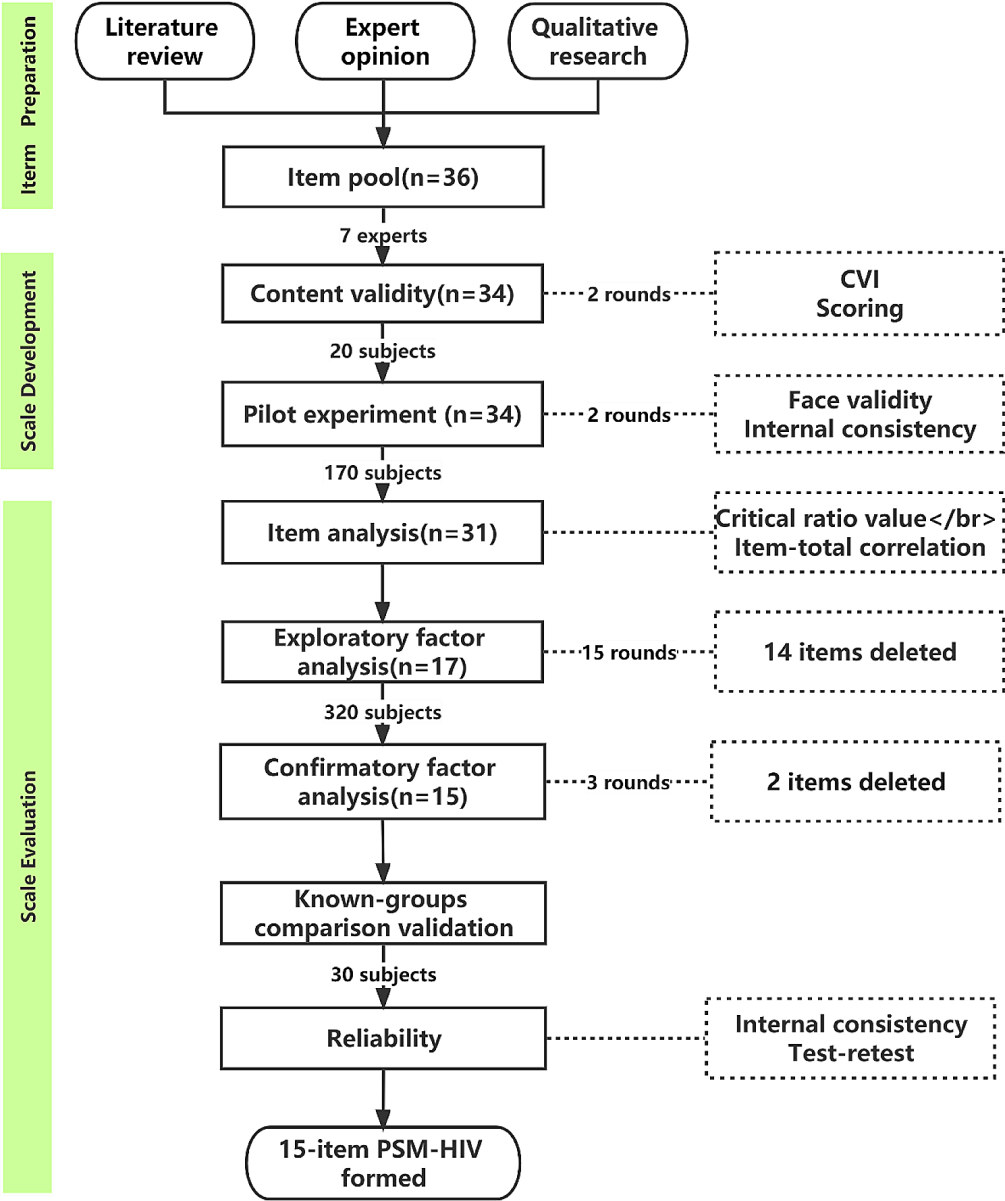
Flow chart in the study CVI: content validity index PSM-HIV: Perceived HIV Symptom Manageability scale

As the primary institution for HIV prevention, treatment, and management in the capital region, Beijing Ditan Hospital receives PLWH from all over the country who are infected with HIV for diagnosis and care. The hospital’s ability to accommodate individuals from diverse backgrounds in terms of gender, ethnic culture, and disease characteristics makes it the preferred research site for this study. All data collection and testing procedures were carried out at the clinic or inpatient ward of Beijing Ditan Hospital in China between June 2021 and September 2021. The overall research process primarily involved item preparation, scale development, and evaluation (Fig. 1). First, item preparation was constructed based on literature review, expert opinions, and the process of qualitative research. Second, Secondly, the scale was initially constructed through two stages: content validity and cognitive interviews (polit experiment). Subsequently, Psychological measurements of the scale were completed through processes such as item analysis, structural validity analysis, reliability analysis, and so on. Both qualitative and quantitative methods were employed in the subsequent stages. Qualitative research was conducted using hermeneutic phenomenology, while the quantitative research component employed a cross-sectional study design.

### Study population

In the qualitative research phase, Criterion sampling was employed, while in the scale testing phase, convenience sampling was utilized. We enlisted several healthcare providers from the clinic or inpatient wards as fled investigators responsible for recruiting participants and gathering data. Individuals meeting the following inclusion criteria were invited to participate in the scale testing: individuals diagnosed with HIV infection or AIDS; Aged 18 and above. Individuals in critical condition or facing a life-threatening situation were excluded. PLWH who gave their consent to participate in the study provided recent CD4 + T cell count laboratory results to the researchers and completed a survey, which included the collection of demographic information, medical history, as well as their emotional responses, self-awareness, attitudes, and behaviors when facing symptoms. Our survey was administered through either an online questionnaire or a paper version. Participants with smartphones could utilize them to complete the online questionnaire, while those without smartphones or facing barriers to their use could opt for the paper version. On-site investigators played a key role in guiding patients to access the online questionnaire system.

### Determine the applicable scope and characteristics of the scale

We determined the applicable scope and characteristics through group discussion: (1) Applicable diseases: HIV infection or AIDS, with no restriction on HIV typing, etc. (2) Applicable population: Adult PLWH. (3) Scale type: A self-evaluation scale (4) Purpose of the scale: To quantitatively evaluate the cognitive and emotional response during individual symptom management. (5) Period: The recall period for this scale is limited to “within the past three months”. (6) Culture: under the influence of Chinese philosophical thought. If using this scale in other cultures, cross-cultural adaptation is necessary.

### Preparation of the item pool

At first, we reviewed all related literature and clarified the concept of PSM. Developers thought that the connotation of this concept mainly involves PLWH’s understanding of their predicament and their perception of resource availability, along with their emotional responses. These aspects serve as the foundation guiding their subsequent approaches to symptom management. It was also integrated into the SSMM-HIV and defined as an inherent crucial psychological factor that can influence the ability of PLWH to practice symptom management and continue to affect patients’ health behaviors, such as compliance with medication. After a preliminary understanding of the concept, to gain a nuanced understanding of this concept, we not only interviewed PLWH but also sought insights from healthcare professionals on how the PLWH comprehend and react to the concept. In-depth interviews with 11 PLWH and focus group interviews with 6 nurses working in HIV wards were conducted. Two well-trained researchers in the research team independently completed the data collection, analysis, and repeated comparison of the results to extract the items’ contents by thematic analysis. After two rounds of group discussion, this pool, which included 36 items, was formed by combining the respondent group’s Chinese cultural background and sensitivity.

### Scoring

The 36-item instrument measures the confidence level and attitude of PLWH to manage their symptoms during the past three months. PLWH rated their position on a five-point Likert scale: 1 = Strongly Disagree, 2 = Disagree, 3 = Generally (neither agree nor disagree), 4 = Agree, 5 = Strongly Agree. Scores on the entire scale range from 34 to 170, with higher scores indicating that PLWH are more confident in managing or controlling symptoms. The research team made all the procedures.

### Scale development

#### Expert consultation

Two rounds of consultation were conducted through email with a panel of seven medical experts with expertise in the management of HIV, psychology, and psychiatry, namely the content validity of the scale, which is evaluated by the item-level content validity index (I-CVI) and the scale-level content validity index (S-CVI). These experts were two nurses with extensive experience taking care of PLWH, three physicians with expertise in treating PLWH, a nursing faculty with expertise in questionnaire development, and a psychological or psychiatric care specialist for many years. Experts needed to score the relevance of each item on a 4-point scale: 1="not relevant at all,” 2=” must be modified, otherwise not relevant,” 3=” applicable to the study, but needs minor revision, ”4="very relevant.” The I-CVI was calculated by dividing the number of experts with scores of 3 and 4 by the total number of experts. The S-CVI is obtained by computing the average of all I-CVIs. S-CVI > 0.90 and I-CVI > 0.78 indicated good content validity [25].

#### Pilot experiment

Twenty subjects who met the inclusion and exclusion criteria were selected for the presurvey. The items’ appropriateness, difficulty, ambiguity, and complexity were evaluated. The inclusion criteria were: confirmed diagnosis of HIV infection according to The Chinese AIDS Diagnosis and Treatment Guidelines (2018 edition); an age of ≥ 18 years; in-patient or out-patient with HIV in designated hospitals of the selected study regions. The exclusion criteria were: participants were unable to complete the questionnaire due to physical or mental health reasons and refused to participate. Based on the strict inclusion and exclusion criteria, participants were invited to participate in the survey. Generally, PSM-HIV scale takes about less than 6 min to administer. At this stage, this instrument was further optimized.

### Scale evaluation

#### Item analysis

The item analysis was performed using the critical ratio (CR) and item-total correlation (ITC) [26] based on 170 subjects’ data that met the inclusion and exclusion criteria as above. After the total scale score was calculated and ranked, according to the critical score of 27%, the subjects were divided into a high group and a low group. After the independent-samples t-test (the significance of the average difference between the high and low groups in each item was calculated), the items that did not reach significance were deleted. The remaining items were then used to be removed according to the ITC < 0.4.

#### Structural validity

Structural validity was evaluated by factor analysis, including exploratory factor analysis (EFA) and confirmatory factor analysis(CFA) [27]. Additionally, convergent validity (CV) and discriminant validity (DV) were added to evaluate the scale’s validity. CV was assessed by factor loading, Composite Reliability (CR), and Average Variance Extracted (AVE) [28]. AVE refers to how much variation explained by potential factors comes from measurement error [28, 29]. The larger the AVE is, the larger the percentage of variation explained by potential factors is, and the smaller the relative measurement error. The average variance extracted (AVE) values > 0.36 and the composite reliability (CR) > 0.70 indicated that the CV of dimensions was acceptable. DV was compared to test the extent to which the scale differed between subgroups with different health conditions in the study sample [26]. Inter-group comparisons of scores of PSM after grouping by characteristics were a standard measure of DV. Based on the factor load value, the Model Fit Summary in the EFA, and other fundamental values, the dimensions and items of the final version of the scale were determined. EFA and CFA were computed based on two different sample size groups within the target population. EFA was analyzed using data from a sample of 170 individuals, while CFA was conducted using data from an additional sample of 320 participants. The selection of subjects was still followed by the same inclusion and exclusion criteria. Moreover, in our study, we assessed the known validity of the group by comparing the differences in scores on the PSM-HIV scale among PLWH at different stages of the disease to further verify the structural validity of this scale.

#### Reliability

Reliability was evaluated using Cronbach’s coefficient alpha (Cronbach’s α), McDonald´s Omega (McDonald’s ω), and the Test-retest. Cronbach’s α and McDonald´s Omega is often used to assess the internal consistency of measuring tools by calculating the average correlation among the scale components. Test-retest reflects the stability of the test over time; the same test method is used to test the same group of subjects twice under the same environment every 10 ∼ 14 days, and then Intraclass Correlation Coefficient (ICC) was calculated using a single-measurement, absolute-agreement, 2-way mixed-effects model to evaluate the retest reliability of the scale. Cronbach’s α or McDonald’s ω>0.73 [30] and ICC>0.75 [31] is acceptable. According to the suggestion that the minimum sample size for retest reliability evaluation is 30, 30 participants met the same inclusion and exclusion criteria in this study, who were hospitalized in the ward and were medically judged to be able to stay for more than a week, finally filled in the scale [31].

### Statistical analysis

Statistical analysis was conducted using SPSS Statistical software (version 20.0, IBM, Chicago, IL) and Amos (version 26, IBM, Chicago, IL). Different methods were used to evaluate this tool, including Cronbach’s α, ICC with a single-measurement, absolute agreement, 2-way mixed-effects model, Kruskal-Wallis analysis of variance, t-test, factor analysis, etc. If there was only one missing item, the mean replaced the score. The scale was excluded from the analysis if ≥ 3 items were left unanswered.

### Ethics approval

Ethical approval was obtained from the Institutional Review Board of Beijing Ditan Hospital Capital Medical University, where the study was conducted, reference number NO. DTEC-KY2021-015-01. We obtained written informed consent from all participants. To ensure the confidentiality of the data, participants first took part in the survey anonymously, and all personal information and data materials were placed separately and managed by special personnel. All experiments were performed according to relevant guidelines and regulations (such as the Declaration of Helsinki).

## Results

### Participant characteristics

In total, 540 PLWH completed the survey and were admitted to the Beijing Ditan Hospital. The process of answering the questions was supervised by one-to-one personnel on-site rather than induced. These participants ranged in age from 19 to 73 years, with an average age of (41.03 ± 10.58)years. Additional detailed demographic information can be found in Table 1.

**Table 1:** Socio-demographics and clinical characteristics of 540 PLWH

Characteristics	Frequency(n)/X ± S	%
Age	41.03 ± 10.583	
Gender		
Male	451	83.5
Female	89	16.5
Education level		
Middle school or below	148	27.4
High school or equivalent	129	23.9
University degree or equivalent	229	42.4
Master’s or above	34	6.3
Employment status		
Student	22	4.1
On the job	213	39.4
Unemployment	66	12.2
Freelancer	195	36.1
Retirement	44	8.2
Living situation		
alone	193	35.7
with family	260	48.2
with friends or classmates	55	10.2
Otherwise	32	5.9
Living Region		
Urban	424	78.5
Rural	116	21.5
Years of HIV diagnosis(years)		
<1	63	11.7
1 ∼ 5	199	36.8
6 ∼ 10	148	27.4
>10	130	24.1
ART use		
Yes	477	88.3
No	63	11.7
Years of ART use(years)		
<1	50	10.5
1 ∼ 5	189	39.6
6 ∼ 10	140	29.4
>10	98	20.5
Disease Staging		
acute infection period	21	3.9
asymptomatic	332	61.5
AIDS	187	34.6

### Measurement properties

#### Initial scale

The initial scale contained 36 items from the literature review (7 items), phenomenological research (25 items), and group discussion (4 items). Through 2 rounds of expert consultation and a pilot experiment, seven experts evaluated the content validity, and 20 participants also gave their opinions on all terms. Content validity was based on the content validity index (CVI). The CVI of a 36-item scale ranged from 0.71 to 1.00, the mean was 0.94, and content coverage reached 87.57% in the first round. After five items were deleted, three items were added, and five items were modified, the CVI of the scale was adjusted to be 0.99 (0.85 ∼ 1.00). A 34-item scale was formed. 20 PLWH completed a pilot experiment to test the reliability of the items, and the response rate was 100%. After 20 PLWH judged the content of the scale from the aspects of appropriateness, difficulty, ambiguity, and complexity, five items were adjusted again. Cronbach’s α for the PSM-HIV scale was 0.96.

#### Item analysis

Item analysis was performed using CR and ITC. It should be noted that items 16,19 and 24 on the scale were reverse-scoring items, so reverse coding was required. Before the analysis, the three items were recoded and assigned, then the total scores of all questions were added and sorted. The score of 170 PLWH was 134.93 ± 18.36(46 ∼ 170).

The high group (top 27%)(157.70 ± 5.49) and the low group (bottom 27%)(113.28 ± 15.42) were selected, i.e., the total score below 128 was the low group and the total score above 147 was the high group. After the independent-samples t-test, all the items that reached significance(*P*<0.01) remained. According to ITC’s criteria, items 6, 16, 19, and 24 were gradually removed step by step until item 6 was left, that is, these ITC values were 0.37, -0.26, -0.32, -0.17, and *P*<0.05, 31 items remained at this stage and correlation of the items, see Table 2.

**Table 2:** Item analysis (ITC)

Pearson correlation	Total score
Item1	0.627**
Item2	0.693**
Item3	0.782**
Item4	0.667**
Item5	0.696**
Item6	0.400**
Item7	0.731**
Item8	0.706**
Item9	0.591**
Item10	0.753**
Item11	0.749**
Item12	0.528**
Item13	0.671**
Item14	0.767**
Item15	0.728**
Item17	0.696**
Item18	0.769**
Item20	0.744**
Item21	0.779**
Item22	0.734**
Item23	0.721**
Item25	0.672**
Item26	0.789**
Item27	0.740**
Item28	0.622**
Item29	0.747**
Item30	0.738**
Item31	0.755**
Item32	0.567**
Item33	0.760**
Item34	0.671**

**. *P*<0 0.01

*. *P*<0.05

#### Exploratory Factor Analysis

This phase went through 14 rounds of exploratory factor analysis (EFA), and 14 items were removed step by step in each round of trials according to the deletion criteria based on the data of 170 PLWH. The Principal component analysis(PCA) was used for factor extraction, and the orthogonal rotation method was used to select the common factor rotation axis [32]. The remaining 17 items of the scale showed better results. The Kaiser-Meyer-Olkin (KMO) index was 0.91>0.9, indicating that the sample was adequate for factor analysis. And Bartlett’s Test of Sphericity was significant (χ2 = 1701.24; df = 136, *P*<0.01) (Table 3), indicating that the relationship among the variables was strong and the data was suitable for an EFA. According to the factor loading recommended by Horn [33], three factors were obtained(Eigenvalues > 1), meaning the number of domains of the scale. A total variance of 63.10% explains the extracted factors. The three factors were named as follows based on the construct of the concept: Factor 1: Cognitive-Behavioral (CB-8), comprising eight items with factor loadings ranging from 0.66 to 0.81. Factor 2: Affective Interaction (AI-5) with five items, and factor loadings ranged from 0.53 to 0.82. Factor 3: Self-Attitude (SA-4) with four items, and factor loadings ranged from 0.56 to 0.78. Table 4 displays factor loadings above 0.50.

**Table 3:** KMO and Bartlett’s Test

Kaiser-Meyer-Olkin value		0.91
Bartlett’s Test	χ2	1701.24
	df	136
	Sig.	0.00

**Table 4:** Results of Cronbach’s α and CFA for PSM-HIV

Facor	Items	Factor loading	Eigenvalue	Cumulative explanatory variance	Cronbach’s α/McDonald’s ω	Correlation coefficient
**1**	**item20**. Even if I have slight adverse reactions after taking medicine, I will still follow the medication plan made by the healthcare practitioners and take medicine regularly.	0.746	8.188	29.136	0.907/0.927	0.935**
	**item4**. I have a clear understanding of which symptoms need professional help to be controlled or alleviated.	0.742				
	**item7**. I trust and follow the knowledge of professional symptom management healthcare practitioners provide.	0.730				
	**item29**. The medication guidance that healthcare practitioners provide can help me cope with adverse drug reactions.	0.698				
	**item8**. I can proactively adjust negative behaviors in my life to reduce the occurrence of symptoms	0.690				
	**item27**.Through learning, I can understand what symptoms may occur in the future.	0.687				
	**item33**. I can easily get consultation or help from medical institutions or social care organizations when symptoms occur.	0.664				
**2**	**item13**.Maintaining a good relationship with relatives or friends and communicating with each other can alleviate the tension and discomfort when my symptoms occur.	0.819	1.503	48.187	0.850/0.901	0.899**
	**item31**.I am encouraged that my experience can help other patients.	0.692				
	**item26**. The care of my family/friends give me the confidence to fight the discomfort caused by symptoms.	0.686				
	**item25**. Communication and interaction with healthcare practitioners can help me control or alleviate the pain caused by symptoms.	0.639				
**3**	**Item6.** When symptoms occur, I care more about my feelings than laboratory findings.	0.784	1.036	63.100	0.711/0.822	0.815**
	**Item34**. I can calmly face the occurrence of common symptoms.	0.772				
	**Item12**. I would still participate in social activities normally when the symptoms are mild.	0.647				
	**Item32**. I think my role in dealing with symptoms is significant (for example, while help and care from professionals or loved ones played a role in combating the symptoms, my positive attitude, hope, and belief in proactively seeking treatment meant more than any other factors.).	0.562				

**. *P*<0 0.01 (double tail), the correlation is significant

#### Confirmatory factor analysis

**Fig. 2:**
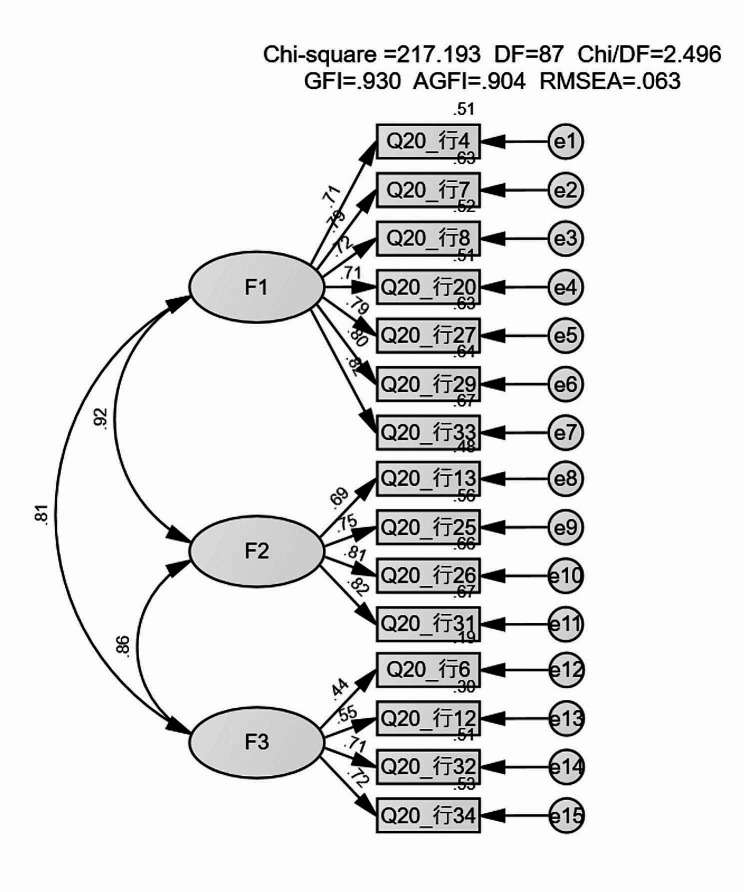
The standardized estimates of each coefficient in the CFA model CFA: confirmatory factor analysis F1, F2 and F3: stand for three different factor Chi-square: χ^2^, Chi/DF: χ^2^/df, GFI: Goodness-of-Fit Index, RMSEA; The value of Root Mean Square Error of Approximation

The Structural validity was further evaluated by three rounds of confirmatory factor analysis (CFA) using data from 320 other subjects. After adjusting according to Modification Indices(MI), items 5 and 28 (numbered as the source scale) were deleted. The model fit of the 15-item scale is relatively stable, and the standardized estimates are presented in Fig. 2. Common-fit indices in the CFA model are shown in Table 5. All the standardized factor loading coefficients obtained were > 0.6 except item 6 = 0.44 < 0.5. All the path coefficients on the three subscales were > 0.5. In addition, Squared Multiple Correlations (SMC) of other items > 0.36 except item 6 and item 12.

**Table 5:** Common-fit indices in the CFA model

Model	CMIN	DF	CMIN/DF	RMR	RMSEA	GFI	AGFI	NFI	IFI		CFI		AIC	
**Default model**	217.19	87	2.50	0.03	0.06	0.93	0.90	0.93	0.96	0.96		283.19		

#### Convergent validity

In this Model, AVE was 0.38 ∼ 0.59, and CR was 0.70 ∼ 0.91, Indicating that the convergent validity of the scale is acceptable (Table 6).

**Table 6:** The value of CR and AVE

Observable Variable	Latent variable	λ(Std)	Estimate (Unstd)	S.E.	*t*-value	*P*	Label	SMC	CR	AVE
item4	F1	0.71	1					0.51	0.91	0.59
item7	F1	0.80	1.08	0.07	14.86	***	par_1	0.63		
item8	F1	0.72	0.98	0.07	13.53	***	par_2	0.52		
item20	F1	0.71	0.89	0.07	13.31	***	par_3	0.51		
item27	F1	0.79	1.14	0.08	14.75	***	par_4	0.62		
item29	F1	0.80	1.02	0.07	14.85	***	par_5	0.64		
item33	F1	0.82	1.08	0.07	15.22	***	par_6	0.67		
item13	F2	0.70	1					0.48	0.85	0.59
item25	F2	0.75	0.94	0.07	13.38	***	par_7	0.56		
item26	F2	0.81	1.04	0.07	14.42	***	par_8	0.66		
item31	F2	0.82	1.01	0.07	14.53	***	par_9	0.67		
item6	F3	0.44	1					0.19	0.70	0.38
item12	F3	0.55	1.12	0.16	6.90	***	par_10	0.30		
item32	F3	0.71	1.23	0.16	7.50	***	par_11	0.51		
item34	F3	0.73	1.30	0.17	7.77	***	par_12	0.53		

***. *P*<0 0.001

#### Known-groups comparison validation

The PSM-HIV scores of PLWH at three different stages, respectively, were 51.85 ± 11.65 (acute infection period), 62.11 ± 8.32 (asymptomatic stage), and 60.94 ± 10.52 (AIDS stage). There are statistical significances among PLWH at different stages of the disease (χ^2^ = 9.02; df = 2, *P*<0.05).

#### Reliability

##### Internal consistency

Cronbach’s α of the PSM-HIV and the three subscales were 0.924, 0.907, 0.850, and 0.711, respectively (Table 4). The values of McDonald’s ω were 0.940, 0.927, 0.901, and 0.822, respectively (Table 4).

##### Test-retest

The ICC of the PSM-HIV was 0.88, with a 95% confidence interval of 0.76 to 0.94, reaching statistical significance (*P* < 0.01). This implies that there is a 95% probability that the true ICC value falls within the range of 0.76 to 0.94. Consequently, based on statistical inference, it would be more accurate to characterize the reliability level as ranging from “good” to “excellent.”

## Discussion

Although symptoms are difficult to control from a medical perspective, supporting patients to gain a sense of symptom manageability is essential to improve overall well-being [34]. Based on the Common Sense Model (CSM) of Leventhal [35], the concept of PSM defined by Fierz [20]and the literature review implied that PSM was related to psychological social and other factors. Meanwhile, understanding perceived manageability might be easy and straightforward for healthcare professionals to support symptom management and reduce symptom-related distress [36]. Hence, it is imperative to consider and evaluate the fundamental significance of this concept in the context of symptom management domains that are specific to PLWH.

Given these, a 15-item scale, “PSM-HIV,” was developed that include three subscales (Cognitive-Behavioral, Affective Interaction, and Self-Attitude), was developed through a literature review, expert opinion, and a phenomenological process. The refinement of all items was based on the ability of the research team and the choice of experts who should have expertise in relative fields and willing to participate in research. In the conceptual analysis of PSM, researchers have emphasized the perceived self-manageability or control of symptoms, which implies certain attitudes and beliefs about self-cognition, self-behavior, and the availability of resources. Self-efficacy theory was widely used in contemporary psychology to study and explain people’s ability to understand their confidence, perception, or belief. The Belief Theory elaborated that a belief was a particular feeling, emotion, or view. On the basis of the above theories, we considered individual subjectivity and resource availability as vital roles in the PSM. Among these three factors, Cognitive-Behavioral and Self-Attitude stand for individual subjectivity, and Affective Interaction stands for the availability of resources.

The response rate and feedback rate of the seven experts was well presented in this study. The critical ratio (CR) and item-total correlation (ITC) in 15 items of the PSM-HIV scale were acceptable, meaning that items have adequate discrimination and correlation. After factor analysis, three factors were extracted according to strict criteria that were very consistent with the Belief Theory [26], Bandura’s self-efficacy theory [27], and the connotation of the PSM [20]. The Kaiser-Meyer-Olkin (KMO) and Bartlett’s Test results also showed they were appropriate for factor analysis. During this process, confirmatory factor analysis (CFA) was performed dozens of times using comprehensive deletion criteria, including that factor loading of an item was greater than 0.4/0.5 on multiple factors at the same time; the factor loading of an item was below 0.4/0.5; there were less than three questions in a factor. Items were deleted step by step and explored repeatedly. After 15 deletion rounds, the dimensions of the clustered items showed a reasonable theoretical explanation and stable distribution.

According to indices of confirmatory factor analysis (CFA) values, the goodness-of-fit indices analyzed in this study suggested adequate fit, χ2/df (CMIN/DF)(2.50<3), Root Mean square Residual (RMR) (0.03<0.05), Goodness-of-Fit Index (GFI) (0.93>0.9), Adjusted Goodness-of-Fit Index (AGFI) (0.90>0.9), Normed Fit Index (NFI) (0.93>0.9), Incremental Fit Index (IFI) (0.96>0.9), Comparative Fit Index (CFI) (0.96>0.9), Akaike Information Criterion (AIC)(283.19). The Root Mean Square Error of Approximation (RMSEA) value was 0.06, ranging from 0.05 ∼ 0.08; based on McDonald’s advice, it was supposed to be a fair fit [37]. However, the standards of RMSEA are not consistent among experts at present [38]. Generally speaking, the range of RMSEA value on a new scale can be more comprehensive according to experts’ experience. All path coefficients in the three subscales showed a high correlation among the factors, but the Squared Multiple Correlations (SMC) values of item 6 and item 12 <0.36. After discussion and analysis by the research group, given the average variance extracted (AVE) of F3 seemed to be lower than others though, within the normal range, the reason may be rooted in the content narration of items 6 and 12, which may partially overlap with items descriptions in other dimensions. Finally, items 6 and 12 were modified as “when symptoms occur, I prefer to believe in my personal feelings, which contain more value” and “If symptoms are mild, I am still willing to participate in normal work, life, and social activities.”.

Internal consistency refers to the degree of inter-relatedness among the items. Cronbach’s α can be used to assess the internal consistency of an outcome measurement instrument (OMI) that is unidimensional by factor analysis; an α value of ≥ 0.70 is required for the excellent quality of OMIs [39]. Cronbach’s α for the three subscales ranged from 0.71 to 0.92. Therefore, the 15-item PSM-HIV was considered to have good internal consistency reliability. Test-retest reliability can reflect the scale’s stability, and the ICC is a more desirable measure of reliability and should reflect both the degrees of correlation and the agreement between measurements [31]. The ICC of the 15-item PSM-HIV was high, indicating good reliability.

## Conclusion

The findings from this preliminary trial indicate that the 15-item PSM-HIV may serve as a valid instrument for assessing PSM in PLWH. However, further replication with a larger and more diverse sample of individuals with HIV is necessary to validate these results. In conclusion, the PSM-HIV scale holds potential as a valuable tool for clinical assessment and future research. Its utilization could enhance healthcare providers’ comprehension of the psychological aspects of symptom management, fostering collaborative efforts between healthcare providers and PLWH to explore optimal strategies for symptom management.

## Data Availability

The datasets generated and analyzed during the current study are not publicly available due to the privacy of people living with HIV. Still, they are available from the corresponding author upon reasonable request.
